# *Anopheles stephensi* Mosquitoes as Vectors of *Plasmodium* *vivax* and *falciparum*, Horn of Africa, 2019

**DOI:** 10.3201/eid2702.200019

**Published:** 2021-02

**Authors:** Fitsum G. Tadesse, Temesgen Ashine, Hiwot Teka, Endashaw Esayas, Louisa A. Messenger, Wakweya Chali, Lisette Meerstein-Kessel, Thomas Walker, Sinknesh Wolde Behaksra, Kjerstin Lanke, Roel Heutink, Claire L. Jeffries, Daniel Abebe Mekonnen, Elifaged Hailemeskel, Surafel K. Tebeje, Temesgen Tafesse, Abrham Gashaw, Tizita Tsegaye, Tadele Emiru, Kigozi Simon, Eyuel Asemahegn Bogale, Gedeon Yohannes, Soriya Kedir, Girma Shumie, Senya Asfer Sabir, Peter Mumba, Dereje Dengela, Jan H. Kolaczinski, Anne Wilson, Thomas S. Churcher, Sheleme Chibsa, Matthew Murphy, Meshesha Balkew, Seth Irish, Chris Drakeley, Endalamaw Gadisa, Teun Bousema

**Affiliations:** Armauer Hansen Research Institute, Addis Ababa, Ethiopia (F.G. Tadesse, T. Ashine, H. Teka, E. Esayas, W. Chali, S.W. Behaksra, D.A. Mekonnen, E. Hailemeskel, S.K. Tebeje, T. Tafesse, A. Gashaw, T. Tsegaye, T. Emiru, E.A. Bogale, G. Shumie, S.A. Sabir, E. Gadisa);; Radboud University Medical Center, Nijmegen, the Netherlands (F.G. Tadesse, L. Meerstein-Kessel, K. Lanke, R. Heutink, E. Hailemeskel, S.K. Tebeje, T. Bousema);; Addis Ababa University, Addis Ababa (F.G. Tadesse, D.A. Mekonnen, E. Hailemeskel);; United States Agency for International Development, Addis Ababa (H. Teka, S. Chibsa, M. Murphy);; London School of Hygiene and Tropical Medicine, London, UK (L.A. Messenger, T. Walker, C.L. Jeffries, K. Simon, C. Drakeley, T. Bousema);; President’s Malaria Initiative VectorLink Ethiopia Project, Addis Ababa (G. Yohannes, P. Mumba, M. Balkew);; Oromia Regional Health Bureau, Adama, Ethiopia (S. Kedir);; President’s Malaria Initiative VectorLink Project, Rockville, Maryland, USA (D. Dengela);; World Health Organization, Geneva, Switzerland (J.H. Kolaczinski);; Liverpool School of Tropical Medicine, Liverpool, UK; (A. Wilson);; Imperial College London, London (T.S. Churcher);; United States President’s Malaria Initiative, Atlanta, Georgia, USA (S. Chibsa, M. Murphy, S. Irish);; Centers for Disease Control and Prevention, Atlanta (S. Irish)

**Keywords:** *Anopheles stephensi*, mosquitoes, urban malaria, vector competence, membrane feeding, emerging, outbreak, transmission, *Plasmodium falciparum*, *Plasmodium vivax*, parasites, Horn of Africa

## Abstract

*Anopheles stephensi* mosquitoes, efficient vectors in parts of Asia and Africa, were found in 75.3% of water sources surveyed and contributed to 80.9% of wild-caught *Anopheles* mosquitoes in Awash Sebat Kilo, Ethiopia. High susceptibility of these mosquitoes to *Plasmodium falciparum* and *vivax* infection presents a challenge for malaria control in the Horn of Africa.

Malaria control programs in Africa traditionally focus on rural settings, although transmission is also a health concern in some urban settings (*1*). *Anopheles stephensi* mosquitoes breed predominantly in urban settings, prefer water storage containers (*2*), and are found throughout the Horn of Africa (*3*). To determine susceptibility of *An. stephensi* mosquito vectors to infection with local *Plasmodium* strains, we measured their abundance in an urban area of Ethiopia and characterized their aquatic habitats, biting and resting behavior, and competence to transmit local *P. vivax* and *P. falciparum*.

Study protocol was approved by the Institutional Ethical Review Board of the Aklilu Lemma Institute of Pathobiology of Addis Ababa University (ALIPB IRB/025/2011/2019), the Oromia Regional Health Bureau (BEFO/MBTFH/1331), and AHRI/ALERT Ethics Review Committee (AF-10-015.1, PO07/19). All participants or parents/legal guardians for participants <18 years of age provided written informed consent. Persons who volunteered for human landing collection also provided written informed consent, were monitored for 3 weeks after collections, and if symptomatic and positive received treatment for *Plasmodium* according to the treatment guidelines of the country.

## The Study 

This study was conducted in Awash Sebat Kilo, Ethiopia, an area of perennial malaria transmission, during April–September 2019. We examined aquatic habitats for immature-stage *Anopheles* mosquitoes by standard dipping (10×/site) for 5 consecutive days (*4*). We assessed mosquito resting, feeding, and host-seeking behavior by 5 methods: CDC miniature light traps model 512 (John W. Hock Company, https://www.johnwhock.com), human landing collection, pyrethrum spray sheet collection, aspiration from animal shelters, and cattle-baited traps (*5*). We identified adult mosquitoes by using standard keys and confirmed identification by targeted sequencing of nuclear internal transcribed spacer 2 (ITS2) and mitochondrial cytochrome oxidase subunit 1 gene (COI) (*6*). To generate clade topologies, we compared *An. stephensi* mosquito DNA sequences with those in publicly available libraries (*7*). We determined mosquito blood meal sources by using multiplex PCR targeting cytochrome b (*8*) and infection status by using 18S rRNA nested PCR (*9*).

Adult *An. stephensi* mosquitoes reared from immature mosquitoes from local water sources and a colony of *An. arabiensis* mosquitoes (≈120 each) were fed in the dark for 30 min on membrane feeders containing fresh blood from Adama malaria clinic patients with microscopy-confirmed mono- and mixed-species infections with *P. vivax* and *P. falciparum* (*10*). Unfed and partially fed mosquitoes were removed; fully engorged mosquitoes were maintained on sugar solution. At 7 or 12 days after feeding, mosquitoes were dissected, their midguts were examined for oocysts, and their salivary glands were examined for sporozoites. To compare infection status between *An. arabiensis* and *An. stephensi* mosquitoes, we performed logistic regression. We used individual mosquito data and a fixed effect for each patient to account for correlations between mosquito observations from the same donor. Bland-Altmann plots were generated for differences in infectivity between mosquito sources by using the Pitman test of difference in variance. For analyses, we used STATA version 13 (StataCorp., https://www.stata.com/company) and GraphPad Prism 5.3 (GraphPad Software Inc., https://www.graphpad.com). Raw data have been deposited in the DRYAD data depository (https://datadryad.org/stash/dataset/doi:10.5061/dryad.gf1vhhmnt).

*An. stephensi* larvae were detected in 75.3% (64/85) of the 85 artificial water sources surveyed (Table 1). A total of 49,393 immature *Anopheles* larvae and pupae were collected during 20 weekly collections in April–September 2019, of which 45,316 (91.7%) emerged as adult mosquitoes in the laboratory. Morphologic identification of adults confirmed that all were *An. stephensi*. During monthly rounds of entomologic surveillance in August and September (6 days each), we collected 89 adult female *Anopheles* mosquitoes (72 [80.9%] *An. stephensi,* 16 *An. gambiae*, and 1 *An. pharoensis*). We detected *P. vivax* in 2.8% (2/72) and *P. falciparum* in 1.4% (1/72) of wild-caught *An. stephensi* mosquitoes. Blood meal source was identified for 35.0% (28/80) blood-fed wild-caught *An. stephensi* mosquitoes; exclusive human blood meal was identified for 17.2% (5/29). The remainder fed (multiple blood meals) either on humans and animals (n = 9) or animals only (n = 14) such as goats (n = 21), cows (n = 4), and dogs (n = 5). Successful sequencing of ITS2 for 76 and COI for 45 *Anopheles* mosquitoes confirmed that all were *An. stephensi*. According to ITS2 sequences, *An. stephensi* mosquitoes from Ethiopia formed a well-supported monophyletic clade with isolates from the Arabian Peninsula and Southeast Asia (Appendix). The COI tree was more resolutive, suggesting that *An. stephensi* mosquitoes from Ethiopia were most closely related to mosquitoes from Djibouti (64%) and Pakistan (54%).

**Table 1:** Characteristics of 85 aquatic habitats surveyed in study of *Anopheles stephensi* mosquitoes as vectors of *Plasmodium* *vivax* and f*alciparum*, Horn of Africa, 2019

Characteristic	Habitats, no.	Mosquito larvae, no. larvae detected/no. habitats sampled (%)	Mosquito pupae, no. pupae detected/no. habitats sampled (%)
Localities (kebeles) within the town of Awash Sebat Kilo			
Sebat Kilo	60	44/60 (73.3)	19/44 (43.2)
Lemlefan	17	12/17 (70.6)	0/12 (0)
Alalamo	8	8/8 (100.0)	5/8 (62.5)
Artificial containers			
Permanent	48	41/48 (85.4)	17/41 (41.5)
Temporary	37	23/37 (62.2)	7/23 (30.4)
Shade status			
Fully	22	14/22 (63.6)	6/14 (42.9)
Partial	24	22/24 (99.7)	7/22 (31.8)
None	39	28/39 (71.8)	11/28 (39.3)
Use status			
In use	71	54/71 (76.1)	20/54 (37.0)
Not in use	14	10/14 (71.4)	4/10 (40.0)
Container material			
Fiber jar/tire	23	10/23 (43.5)	4/10 (40.0)
Metal/steel tanks/drum/barrel	17	16/17 (94.1)	5/16 (31.3)
Cement/ceramic	45	38/4 (84.4)	15/38 (39.5)
Water turbidity			
Clean	56	45/56 (80.4)	17/45 (37.8)
Turbid	28	19/28 (67.9)	7/19 (36.8)

**Figure 1:**
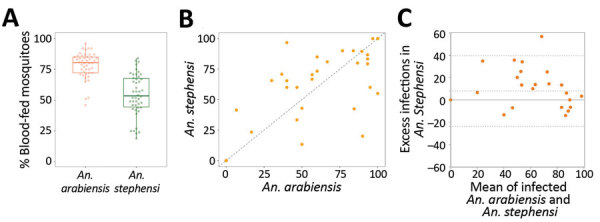
Comparison of feeding efficiency and infection rates for *Anopheles stephensi* and *An. arabiensis* mosquitoes in paired feeding experiments in study of *An. stephensi* mosquitoes as vectors of *Plasmodium vivax* and f*alciparum*, Horn of Africa, 2019. A) Percentage of fully fed *An. arabiensis* mosquitoes (red) and *An. stephensi* mosquitoes (green). Box plots indicate median (midline), 25th (lower line), and 75th (upper line) percentiles of proportion of blood-fed mosquitoes. Whiskers indicate lower and upper 25% scores. Vertical lines indicate minimum and maximum values. B) Percentage of infected mosquitoes. C) Bland-Altman plot (difference plots) for mosquito infection rates in different mosquito species. Symbols indicate differences in infection rates in *An. stephensi* versus *An. arabiensis* (y-axis) mosquitoes in relation to mean infection rates in these 2 species (x-axis). Positive values (57.1%; 16/28) indicate a higher infection rate in *An. stephensi* mosquitoes; dotted lines indicate the 95% limits of agreement. There was no evidence that the correlation coefficient between the paired differences and means differed significantly from 0 (Pitman test of difference in variance, r = 0.026, p = 0.864).

**Figure 2:**
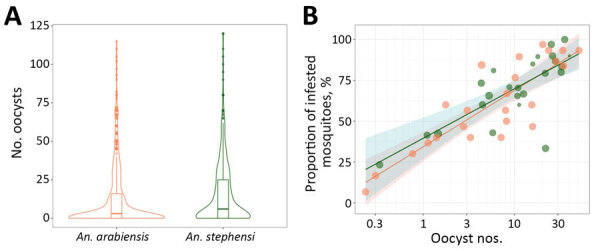
Comparison of relative oocyst numbers and infection rate for *Anopheles stephensi* and *An. arabiensis* mosquitoes in paired feeding experiments in study of *An. stephensi* mosquitoes as vectors of *Plasmodium vivax* and f*alciparum*, Horn of Africa, 2019. Number of oocysts per infected midgut for individual mosquitoes of each of the 2 species. A) Violin plot showing estimated kernel density. Horizontal lines indicate median; box indicates interquartile range; and spikes indicate upper and lower adjacent values. The proportion of midguts with detectable oocysts (y-axis) is indicated in association with log_10_ transformed oocyst numbers (x-axis) for *An. stephensi* (green dots) and *An. arabiensis* (orange dots) mosquitoes. B) Data for 24 feeding experiments in which 723 *An. arabiensis* and 643 *An. stephensi* mosquitoes were dissected. Shaded area indicates 95% CI around estimates for *An. stephensi* (green) and *An. arabiensis* (orange) mosquitoes.

We conducted 47 paired-membrane feeding experiments by using blood from patients with microscopy-confirmed *P. vivax* or *P. falciparum* infection (Table 2). The proportion of blood-fed mosquitoes was generally higher for *An. arabiensis* (median 80.5%; interquartile range [IQR] 72.5–85.0) than *An. stephensi* mosquitoes (median 53.5%, IQR 44.0–68.0; p<0.001; Figure 1, panel A). The proportions of the 2 mosquito species infected with *P. vivax* were strongly associated (ρ = 0.82, p<0*.*001; Figure 1, panel B); a significantly higher proportion of *An. stephensi* (median 75.1%, IQR 60.0–85.9) than *An. arabiensis* mosquitoes were infected (median 58.4%, IQR 40.0–85.6; p<0.042). Allowing for the number of dissected mosquitoes for each set of paired feeding experiments, the odds of an individual mosquito becoming infected was higher for *An. stephensi* mosquitoes (odds ratio [OR] 1.99, 95% CI 1.52–2.59; p<0.001) (Figure 1, panel C). The number of oocysts per infected midgut was also higher for *An. stephensi* (median 17, IQR 6–33) than *An. arabiensis* mosquitoes (median 13, IQR 4–30); p<0.001 (Figure 2, panel A). The number of oocysts was positively associated with the proportion of infected mosquitoes for *An. stephensi* (ρ = 0.553, p<0.001) and *An. arabiensis* mosquitoes (ρ = 0.576, p<0.001; Figure 2, panel B). Among paired feedings, sporozoites were detected in 52.2% (47/90) *An. arabiensis* and 75.0% (84/112) *An. stephensi* mosquitoes. A much higher proportion of *An. stephensi* (51.8%, 58/112) than *An. arabiensis* mosquitoes (31.1%, 28/90) had high sporozoite load (+3 and +4); p = 0.011. After accounting for the number of examined salivary glands, the odds of detecting high sporozoite intensity were substantially higher for *An. stephensi* than *An. arabiensis* mosquitoes (OR 4.6, 95% CI 2.2–9.9; p<0.001).

**Table 2:** Characteristics of blood meals and mosquito feeding outcomes in study of *Anopheles stephensi* mosquitoes as vectors of *Plasmodium* *vivax* and f*alciparum*, Horn of Africa, 2019*

Characteristic	*Plasmodium* species		
	*P. vivax*, n = 36	*P. falciparum*, n = 7	Mixed, n = 4
Parasites/µL, median (IQR)	7,783 (3,603–13,440)	2,431 (867–8,756)	4,516 (1,589–10,563)
Gametocyte positivity, no. positive/no. sampled (%)	25/34 (73.5)	1/7 (14.3)	1/4 (25.0).
Infectious feeds, no. positive/no. sampled (%)	26/36 (72.2)	1/7 (14.3)	2/4 (50.0)
Infected *An. stephensi *mosquitoes, no. positive/no. sampled (%)	446/849 (52.5	2.2	36/104 (34.6)
Infected *An arabiensis *mosquitoes, no. positive/no. sampled (%)	452/1,000 (45.2)	18/200 (9.0)	45/122 (36.9)
Oocysts in infected *An. arabiensis* mosquito midgut, mean (range)	22.8 (1–115)	NA	3.1 (1–22)
Oocysts in infected *An. stephensi* mosquito midgut, mean (range)	24.1 (1–105)	NA	2.8 (1–13)

*Parasite and gametocyte densities were determined by microscopy; IQR, Interquartile range; NA, not available.

## Conclusions

*An. stephensi* mosquitoes have spread from Asia throughout the Horn of Africa, detected in Djibouti in 2012 (*11*), Ethiopia in 2016 (*12*), and Sudan in 2019 (*3*). The widescale presence of *An. stephensi* mosquitoes in developmental stages in artificial water bodies demonstrates that these mosquitoes are firmly established in an urban setting in Ethiopia, located on the main transportation corridor from Djibouti to Addis Ababa. Detection of 4 haplotypes suggests independent arrival of different populations or heterogeneity arising after importation of the mosquito species. Our mosquito feeding experiments predominantly included highly infective patients with clinical *P. vivax* infection (*10*,*13*). Although feeding rates for the membrane-adapted colony of *An. arabiensis* mosquitoes were high, mosquito infection rates were significantly higher for *An. stephensi* mosquitoes*.* Our detection of salivary gland sporozoites establishes that sporogonic development of local *P. vivax* can be completed by *An. stephensi* mosquitoes. We recruited fewer patients with clinical *P. falciparum* infection, who were less likely than *P. vivax* patients to infect mosquitoes (*10*). Despite a modest number of observations, our findings demonstrate that local *P. falciparum* isolates are also capable of infecting *An. stephensi* mosquitoes and are further supported by detection of *P. falciparum*– and *P. vivax–*infected wild-caught adult mosquitoes. 

Spread of *An. stephensi* mosquitoes poses risk for increased *P. falciparum* and *P. vivax* receptivity and local transmission in urban Africa. Given mosquito preference for human-made containers (*14*), our findings support integrated vector management recommended by the World Health Organization under the Global Vector Control Response (*15*). Management may include integrated surveillance and control of other vectors such as *Aedes aegypti* mosquitoes for larval source management.

Appendix Supplemental methods and results for study of *Anopheles stephensi* mosquitoes as vectors of *Plasmodium* *vivax* and f*alciparum*, Horn of Africa, 2019.
