# Supplementary material for: Anopheles stephensi Mosquitoes as Vectors of Plasmodium vivax and falciparum, Horn of Africa, 2019
Source: Emerg Infect Dis. 2021 Feb;27(2):603–7. doi: 10.3201/eid2702.200019 (PMC7853561; doi:10.3201/eid2702.200019)
Supplement: Appendix — Supplemental methods and results for study of Anopheles stephensi mosquitoes as vectors of Plasmodium vivax and falciparum, Horn of Africa, 2019. [file 20-0019-Techapp-s1.pdf]

Article DOI: https://doi.org/10.3201/eid2702.200019

Anopheles stephensi Mosquitoes as Vectors of *Plasmodium vivax* and *falciparum*, Horn of Africa

Appendix

Aquatic habitat characterization

A total of (n = 85) potential larval habitats were surveyed in 5 days (May 2019) in Awash Sebat Kilo town (Appendix Figure 1) using a standardized check list/questionnaire (22 question sets) to capture important characteristics. Immatures were collected throughout the day. Altitude, latitude, longitude, sun light exposure, water turbidity, substrate type, presence of vegetation, predators, and competitors were recorded for each site. The *Anopheles* larvae were separated from culicine larvae and classified as early- (1st, 2nd) or late-instars (3rd and 4th) stage and larval density was recorded by instars. The *Anopheles* larvae/pupae were transported to Adama malaria center with jars and transferred to larval tray for rearing to adult using the same filtered water from the breeding site. Detailed outcome of the aquatic habitat survey can be obtained using this link (https://datadryad.org/stash/dataset/doi:10.5061/dryad.gf1vhhmnt; Table 1). Aquatic habitat characterization) with number of larvae/pupae collected in each of the 10 dips (https://doi.org/10.5061/dryad.gxd2547hr; Table 2_Larvae pupae density).

Adult mosquitoes resting, biting and host preference behavior

Resting, feeding and host preference behavior of *An. stephensi* was assessed using 5 entomological sampling techniques: i) Centers for Disease Control (CDC) light trap, ii) human landing catches (HLC), iii) pyrethrum spray sheet collection (PSC), iv) aspiration from animal shelters, and v) cattle baited traps. The CDC light traps (Model 512; John W. Hock Company, Gainesville, FL, USA) were set 1 m above the ground on a wall or roof, both indoors and

outdoors on 15 randomly selected households for two nights that makes a total of 60 traps in 30 nights. Indoor traps were hung at the foot edge of the person who slept under an untreated bed net (*1*). Other occupants in the houses were left to use LLINs provided by the control program as part of the routine malaria control. The traps were switched on at 6:00PM and off at 6:00AM the next morning in each sampling night. PSC were conducted from 6:00 AM to 2:00 AM on five randomly selected households' per-day and 20 households were included in each round of sampling, thus a total of 60 households were sampled in three rounds. The HLC were conducted in nine selected households both in and outdoors that was repeated the next day. Locally trained entomology technicians were employed to collect female *Anopheles* by standard mouth aspirator from 6:00 PM to 6:00 AM from both indoors and outdoors. Two collectors were assigned at a time for each house (one outdoors and one indoors) in shifts of 6 hours (the first shift being 6:00PM – 12:00PM and the second from 12:00 PM to 6:00 AM). Collectors in the same shift changed with each other between outdoors and indoors every hour after recording their findings on the checklist to avoid bias due to individual variation in attraction and competence. In addition to this, animal sheds were inspected using HC and cattle bait trap (*2*) were conducted for collecting mosquitos biting and resting in animal shelters. Mosquitoes resting in animal shelters and cattle bait traps were collected using standard mouth aspirator for 30 minutes in each, from 5:30 AM to 6:00 AM. All collected *Anopheles* mosquitoes were counted and sorted out morphologically to species level (*3*,*4*) and by their abdominal stage into unfed, freshly fed, half-gravid or gravid (*5*), except those collected by HLC. In animal shelter with high number of mosquito collection was repeated the next morning.

Of the five methods used for mosquito collection in the 2 monthly studies of 6 days each and therefore 12 days in total, *Anopheles* were caught only by the three methods: CDC light traps, aspiration from animal shelter (hand collection), and human landing catches. This makes it an average of 2.5 female *Anopheles* caught per trap per night. Detailed survey results for different adult catch methods is provided using this link (https://datadryad.org/stash/dataset/doi:10.5061/dryad.gf1vhhmnt; Table 3_Adult mosquitoes surveillance).

Optimization of feeding efficiency of adult *An. stephensi* raised from wild collected larvae/pupae

Optimum starvation time for the *An. stephensi* raised from wild collected larvae and pupae was assessed in three different experiments with 2, 3, 4 or 5 hours before feeding evaluated in each experiment (Appendix Table 1).

Appendix Table 1. Feeding optimization for *Anopheles stephensi* raised from wild collected larvae/pupae

Code of Experiment	Starvation hour prior feeding	Number of fed mosquitoes	Feeding efficiency, % of blood fed
E01–01	5	58	39.2
E01–02	4	68	45.9
E01–03	3	85	53.8
E01–04	2	86	50.3
E02–01	5	24	36.4
E02–02	4	31	42.5
E02–03	3	29	44.6
E02–04	2	17	28.3
E03–01	5	33	36.2
E03–02	4	46	48.9
E03–03	3	44	51.6
E03–04	2	35	35.0

Molecular detection of parasites and blood meal sources and targeted sequencing of morphologically identified *An. stephensi* mosquitoes

Plasmodium infection status of individual wild-caught morphologically-confirmed adult *An. stephensi* mosquitoes was assessed using nested polymerase chain reaction (nPCR) targeting the small 18S subunit (*6*) using genomic DNA extracted from homogenate of mosquito's head-thorax and abdomen separately (*7*), indicating sporozoite and oocyst-stage infections, respectively. Multiplex PCR that targets the mitochondrial cytochrome b gene and produces species-specific fragments of varying sizes was used to assess blood meal sources of individual mosquitoes (*8*). For confirmation of morphologically identified *An. stephensi*, DNA was extracted from whole mosquito bodies using the DNeasy Blood and Tissue kit (Qiagen, UK). PCR was performed for each individual mosquito, targeting the nuclear internal transcribed spacer 2 region (ITS2) and the mitochondrial cytochrome oxidase subunit 1 gene (COI) (*9*). Following PCR clean-up (Source BioScience Plc, Nottingham, UK), chain termination sequencing was performed to generate unambiguous consensus sequences for each sample (Supplemental notes). Sequences were assembled manually in BioEdit v7.2.5 (*10*) to create unambiguous consensus sequences for each sample. Consensus sequence alignments per gene were generated in ClustalW and used to perform nucleotide BLAST (NCBI) database queries (*11*). *An. stephensi* ITS2 and COI sequences, from across the vector's geographic range, were

downloaded from GenBank for phylogenetic analysis in MEGA X (*12*). Additional outgroup ITS2 sequences were retrieved for *An. maculatus, An. maculipalpis, An. sawadwongporni* and *An. willmori.* Alternate maximum-likelihood (ML) phylogenies were constructed using the Jukes-Cantor (ITS2; final tree *ln*L = −916.913) or Tamura-Nei (COI; final tree *ln*L = −732.248) models, following appropriate nucleotide substitution model selection in MEGA X. Bootstrap support for clade topologies was estimated following the generation of 1,000 pseudoreplicate datasets. As indicated in Appendix Figure 2. *An. stephensi* from Ethiopia are more related with those from Pakistan and Djibouti.

Sporozoite quantification

Sporozoites were quantified on day 12 post feeding in salivary glands of mosquitoes that remained from the batch where high oocysts were detected during midgut dissection on day 7 post feeding and categorized into four (with a grade from 1–4) following protocol reported before (*14*). Table 4_Sporozoite quantification, following this link (https://datadryad.org/stash/dataset/doi:10.5061/dryad.gf1vhhmnt) depicts the detail results for each *An. arabiensis* and *An. stephensi* dissected (rows) with representative pictures in Appendix Figure 3, panels A, B.

References

1. Lines J, Curtis C, Wilkes T, Njunwa K. Monitoring human-biting mosquitoes (Diptera: Culicidae) in Tanzania with light-traps hung beside mosquito nets. Bull Entomol Res. 1991;81:77–84. https://doi.org/10.1017/S0007485300053268

2. St Laurent B, Oy K, Miller B, Gasteiger EB, Lee E, Sovannaroth S, et al. Cow-baited tents are highly effective in sampling diverse Anopheles malaria vectors in Cambodia. Malar J. 2016;15:440. PubMed https://doi.org/10.1186/s12936-016-1488-y

3. Das B, Rajagopal R, Akiyama J. Pictorial key to the species of Indian anopheline mosquitoes. Zoology. 1990;2:32.

4. Gillies M, Coetzee M. A supplement to the Anophelinae of Africa South of the Sahara. Publ S Afr Inst Med Res. 1987;55:1–143.

5. World Health Organization. Malaria entomology and vector control: Geneva: The Organization; 2003.

6. Snounou G, Viriyakosol S, Zhu XP, Jarra W, Pinheiro L, do Rosario VE, et al. High sensitivity of detection of human malaria parasites by the use of nested polymerase chain reaction. Mol Biochem Parasitol. 1993;61:315–20. PubMed https://doi.org/10.1016/0166-6851(93)90077-B

7. Schriefer ME, Sacci JB Jr, Wirtz RA, Azad AF. Detection of polymerase chain reaction-amplified malarial DNA in infected blood and individual mosquitoes. Exp Parasitol. 1991;73:311–6. PubMed https://doi.org/10.1016/0014-4894(91)90102-3

8. Kent RJ, Norris DE. Identification of mammalian blood meals in mosquitoes by a multiplexed polymerase chain reaction targeting cytochrome B. Am J Trop Med Hyg. 2005;73:336–42. PubMed https://doi.org/10.4269/ajtmh.2005.73.336

9. Folmer O, Black M, Hoeh W, Lutz R, Vrijenhoek R. DNA primers for amplification of mitochondrial cytochrome c oxidase subunit I from diverse metazoan invertebrates. Mol Mar Biol Biotechnol. 1994;3:294–9. PubMed

10. Hall TA. BioEdit: a user-friendly biological sequence alignment editor and analysis program for Windows 95/98/NT. Nucleic Acids Symp Ser. 1999:41:95–8.

11. Altschul SF, Gish W, Miller W, Myers EW, Lipman DJ. Basic Local Alignment Search Tool. J Mol Biol. 1990;215:403–10. PubMed https://doi.org/10.1016/S0022-2836(05)80360-2

12. Kumar S, Stecher G, Li M, Knyaz C, Tamura K. MEGA X: Molecular Evolutionary Genetics Analysis across computing platforms. Mol Biol Evol. 2018;35:1547–9. PubMed https://doi.org/10.1093/molbev/msy096

13. Carter TE, Yared S, Gebresilassie A, Bonnell V, Damodaran L, Lopez K, et al. First detection of *Anopheles stephensi* Liston, 1901 (Diptera: culicidae) in Ethiopia using molecular and morphological approaches. Acta Trop. 2018;188:180–6. PubMed https://doi.org/10.1016/j.actatropica.2018.09.001

14. Joshi D, Choochote W, Park MH, Kim JY, Kim TS, Suwonkerd W, et al. The susceptibility of *Anopheles lesteri* to infection with Korean strain of *Plasmodium vivax*. Malar J. 2009;8:42. PubMed https://doi.org/10.1186/1475-2875-8-42

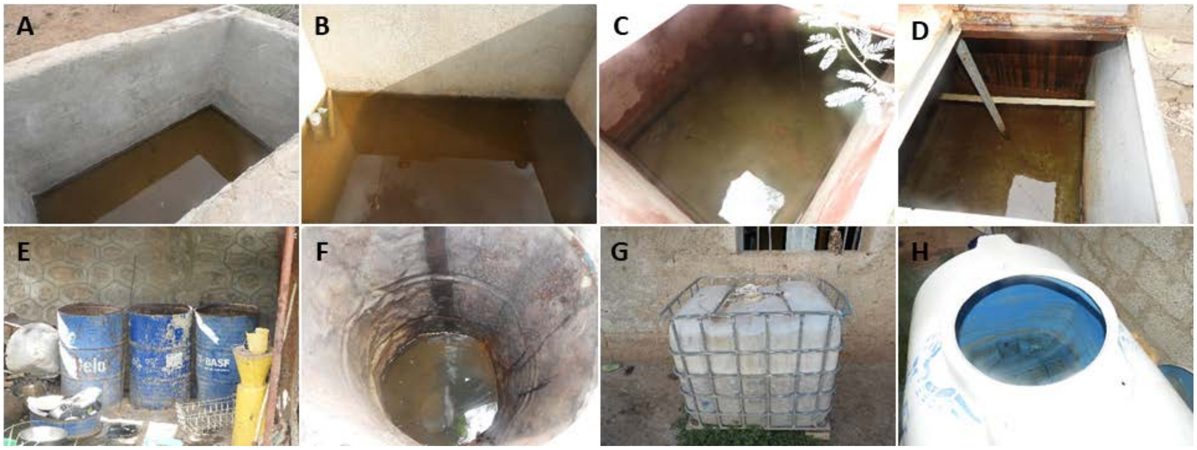

Appendix Figure 1. *Anopheles. stephensi* larval habitats. Images are of waterbodies that were infested with developmental stages of *An. stephensi,* namely water reservoirs made of bricks or cemented tanks (A – B), metal (C and D), barrels (E – F), plastic (G), or fiber (H). The median volume of the aquatic containers was 4m^3 (interquartile range, 1.0–15.6) and ranged from 0.06 m^3 to 360 m^3. The majority of the containers were uncovered and were in use for household (32) and construction purposes (34). The material from which the different types of reservoirs were made of included cement (n = 45), plastic (n = 9), fiber (n = 14) and steel (n = 17).

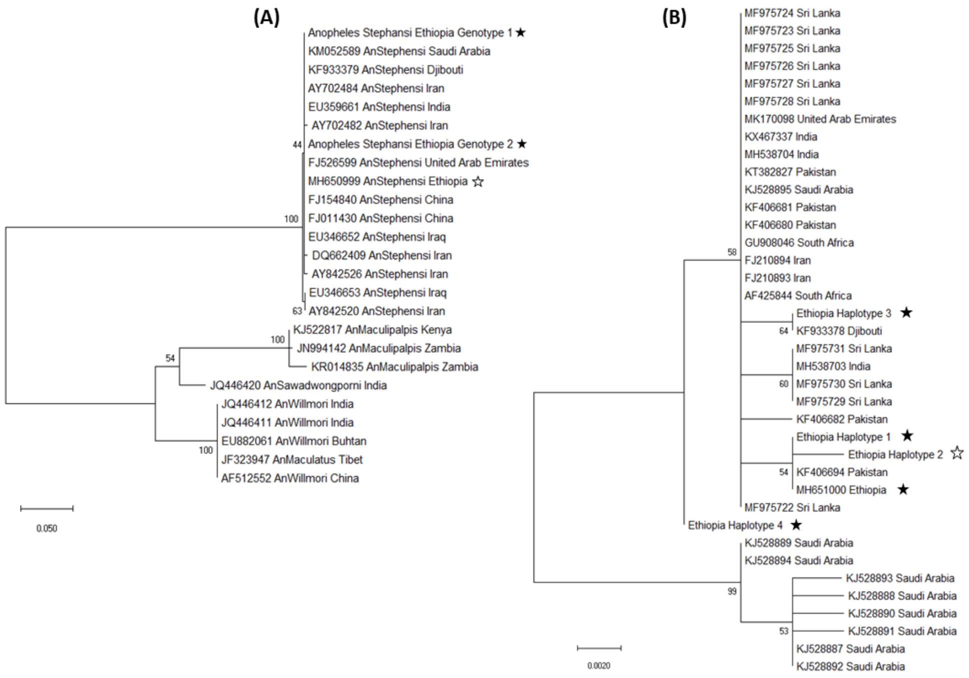

Appendix Figure 2. Maximum-likelihood phylogenies of ITS2 (left) and COI (right). Maximum-likelihood topologies were constructed using representative reference sequences with published geographic data downloaded from GenBank. Within the Ethiopian population, due to the presence of a hyper-variable

microsatellite region, ITS2 sequences (A) were trimmed to create a consensus alignment of 289 bp; one polymorphic site separated samples into two genotypes (indicated with filled asterisk together with the previously reported genotype, MH650999, Carter, et al. (*13*) in unfilled asterisk). COI sequences (B) were assembled into a consensus alignment of 687 bp; a total of four variable sites were identified, corresponding to four haplotypes (indicated with filled asterisk together with the previously reported genotype, MH651000, Carter, et al. (*13*), unfilled asterisk). Nucleotide sequences for ITS2 and COI were deposited in GenBank under the following accession numbers: Ethiopia Genotype1, MN826065; Ethiopia Genotype2, MN826066; Ethiopia Haplotype1, MN826067; Ethiopia Haplotype2, MN826068; Ethiopia Haplotype3, MN826069; and Ethiopia Haplotype4, MN826070. Scale bars indicate nucleotide substitutions per site.
